# The Immune Landscape of Thyroid Cancer in the Context of Immune Checkpoint Inhibition

**DOI:** 10.3390/ijms20163934

**Published:** 2019-08-13

**Authors:** Gilda Varricchi, Stefania Loffredo, Giancarlo Marone, Luca Modestino, Poupak Fallahi, Silvia Martina Ferrari, Amato de Paulis, Alessandro Antonelli, Maria Rosaria Galdiero

**Affiliations:** 1 Department of Translational Medical Sciences (DISMET), University of Naples Federico II, 80131 Naples, Italy; 2 Center for Basic and Clinical Immunology Research (CISI), University of Naples Federico II, School of Medicine, 80131 Naples, Italy; 3 WAO Center of Excellence, 80131 Naples, Italy; 4 Department of Public Health, University of Naples Federico II, 80131 Naples, Italy; 5 Department of Clinical and Experimental Medicine, University of Pisa, School of Medicine, 56126 Pisa, Italy

**Keywords:** angiogenesis, chemokines, dendritic cells, CXCL8, lymphangiogenesis, macrophages, mast cells, neutrophils, thyroid cancer, T reg cells

## Abstract

Immune cells play critical roles in tumor prevention as well as initiation and progression. However, immune-resistant cancer cells can evade the immune system and proceed to form tumors. The normal microenvironment (immune cells, fibroblasts, blood and lymphatic vessels, and interstitial extracellular matrix (ECM)) maintains tissue homeostasis and prevents tumor initiation. Inflammatory mediators, reactive oxygen species, cytokines, and chemokines from an altered microenvironment promote tumor growth. During the last decade, thyroid cancer, the most frequent cancer of the endocrine system, has emerged as the fifth most incident cancer in the United States (USA), and its incidence is steadily growing. Inflammation has long been associated with thyroid cancer, raising critical questions about the role of immune cells in its pathogenesis. A plethora of immune cells and their mediators are present in the thyroid cancer ecosystem. Monoclonal antibodies (mAbs) targeting immune checkpoints, such as mAbs anti-cytotoxic T lymphocyte antigen 4 (anti-CTLA-4) and anti-programmed cell death protein-1/programmed cell death ligand-1 (anti-PD-1/PD-L1), have revolutionized the treatment of many malignancies, but they induce thyroid dysfunction in up to 10% of patients, presumably by enhancing autoimmunity. Combination strategies involving immune checkpoint inhibitors (ICIs) with tyrosine kinase (TK) or serine/threonine protein kinase B-raf (BRAF) inhibitors are showing considerable promise in the treatment of advanced thyroid cancer. This review illustrates how different immune cells contribute to thyroid cancer development and the rationale for the antitumor effects of ICIs in combination with BRAF/TK inhibitors.

## 1. Introduction

Thyroid cancer (TC) is the most frequent type of cancer of the endocrine system [1], accounting for approximately 90% of the endocrine malignancies and for 70% of deaths due to endocrine cancers [2]. During the past two decades, TC incidence increased [3,4]. Risk factors for TC include being female, having a history of goiter or thyroid nodule, a family history of TC, a low-iodine diet, radiation exposure, and obesity [5]. TC is three to four times more likely to develop in women, but it is more aggressive in men, who present with more advanced disease and have lower survival rates [6]. Differentiated TC (DTC) includes papillary thyroid carcinoma (PTC) (≈80%) and follicular thyroid carcinoma (FTC) (≈2%) and medullary thyroid cancer (MTC) (≈4%). Poorly differentiated TC (PDTC) and anaplastic thyroid carcinoma (ATC) account for approximately 5–10% of all TCs [7], but the majority of deaths for TC are attributable to this disease [8,9,10]. Calcitonin-producing parafollicular cells within the thyroid can give rise to MTC [11]. The association between smoldering inflammation and TC has long been recognized [12,13,14]. Indeed, a mixture of immune cells and proinflammatory mediators has been associated with TC initiation and progression [13]. In this review, we discuss recent findings investigating how the cells of the innate and adaptive immune system are involved in TC development and progression. We also discuss the rationale and preliminary results of treatment with monoclonal antibodies (mAbs), targeting immune checkpoints in patients with aggressive TCs.

## 2. Molecular and Genetic Factors

Genetic studies of PTCs reported a high frequency of activating somatic alterations of genes encoding effectors in the mitogen-activated protein kinase (MAPK) signaling pathway, including point mutations of BRAF (≈60%) and the GTPase RAS family genes (≈13%) [15,16,17], as well as fusions involving the REarranged during Transfection *(RET*) [18] and neutrotrophic receptor tyrosine kinase 1 (NTRK1) [19]. The most prevalent (≈60%) mutation in PTC is the activating *BRAF^V600E^* mutation [9,17,20]. The second most prevalent genetic event in PTC is paracentric inversions of the long arm of chromosome 10, causing the fusion of the REarranged during Transfection (RET) intracellular domain and leading to ligand-independent RET kinase activation [21]. RET fusions are found in approximately 7% of sporadic PTC [1] and in about 30% of young patients [22]. Mutations in the tumor suppressor *TP53* gene are the most frequent genetic alterations in PDTC [23]. RAS mutations have also been found in PDTC [24]. Apart from the above-mentioned genetic alterations, several other genes have been found mutated in FTC, including *PI3CA*, *PTEN*, *DICER1*, *EXH1*, and *SPO*P [25,26]. MTC are commonly associated with *RET* mutations [21]. In a minority of PTCs, *EWSR1* rearrangements have been identified [27,28].

The TElomerase Reverse Transcriptase (*TERT*) [29] promoter mutations chr5, 1,295,228 C>T (C228T) and 1,295,250 C>T (C250T) are present, on average, 0% (benign thyroid tumor), 11.3% (PTC), 17.1% (FTC), 43.2% (PDTC) and 40.1% (ATC) in thyroid tumors, displaying an association with aggressive types of TC. Coexisting *BRAF^V600E^* and *TERT* promoter mutations have a robust synergistic impact on the aggressiveness of PTC [30].

ATC is characterized by the accumulation of several different genetic alterations [9]. ATCs and to a lesser extent PDTC are a challenge for genomic studies due to their extensive infiltration of macrophages [31,32]. Three recent studies using a next-generation sequencing [33] approach performed extensive cancer gene exome sequencing in PDTC and ATC [34,35,36]. Profound genomic differences between the two advanced forms of the disease have been found. ATCs harbored a higher number of oncogenic alterations than PDTCs, and the mutations in PDTCs were increased compared to PTC. *BRAF^V600E^* mutations were present in 33% of PDTC and 45% of ATC, whereas mutations in *NRAS*, *HRAS*, or *KRAS* occurred in 28% and 24% of PDTCs and ATCs, respectively. *TERT* promoter mutations, which are important in tumorigenesis, were highly prevalent in ATC compared to PDTC (73% versus 8%). Mutations in *PIK3CA* and *PTEN* were prevalent in ATC (18% and 53%, respectively). Mutations in *EIF1AX* were reported in 11% of PDTC and 9% of ATC. Mutations of genes encoding the phosphoinositide-3-kinase–protein kinase B/*Akt*-mammalian target of rapamycin (PI3K-PKB/*Akt-mTOR*) pathway were more frequent in ATC than PDTC (39% versus 11%). Anaplastic lymphoma kinase (*ALK*) gene rearrangements have been found in ATC [37] and in PTC [38]. *GLIS3* rearrangements are prevalent in hyalinizing trabecular tumor (HTT), which is a rare TC with a characteristic trabecular growth pattern and hyalinization [39]. Collectively, these results have identified several genetic lesions that distinguish PDTC from ATC.

## 3. Cytokines

Cytokines, produced by thyroid-infiltrating immune cells and by follicular cells, play a role in the pathogenesis of autoimmune thyroid disease [14] and contribute to several aspects of TC initiation and growth [13]. Interleukin (IL)-1 stimulated thyroid cell proliferation and the production of IL-8 [40]. IL-1β serum concentrations were increased in patients with atrophic thyroiditis and decreased in those with PTC [41].

IL-4 and IL-13 produced by human T helper 2 (Th2) cells, basophils, and T follicular helper cells (Tfh) [42,43] induce alternative (M2) activation of macrophages and tumor-associated macrophages [44]. Macrophages in TC displayed an M2-like phenotype [45]. The exposure of thyreocytes to ionizing radiation (IR) caused IL-13 production, which stimulated reactive oxygen species (ROS) increase, and was presumably responsible for genetic instability and the emergence of neoplastic clones in TC [46].

TC-derived conditioned media (CM) induced the up-regulation of IL-6 in human mast cells [47] and contributed to epithelial-to-mesenchymal transition (EMT) and stemness, which are features of TC [48]. Studies comparing levels of circulating IL-6 among TC patients and controls have provided inconsistent results [49,50,51].

Tumor-associated macrophages (TAMs) [44] and tumor cells themselves produce IL-10 [52]. IL-10 circulating levels were higher in PTC associated with multinodular goiter (MNG) compared to MNG alone [53]. Malignant epithelial cells isolated from PTC and ATC [7] produced in vitro IL-4 and IL-10, which increased the expression of the anti-apoptotic proteins B-cell lymphoma 2 (Bcl-2), B-cell lymphoma-extra large (Bcl-xL), cellular FLICE (FADD-like IL-1β-converting enzyme)-inhibitory protein (cFLIP), and Phosphoprotein enriched in diabetes/Astrocytic phosphoprotein (PED/PEA-15) in TC cells [54,55]. IL-4 and IL-10 serum concentrations were higher in PTC patients compared to Hashimoto’s thyroiditis [56]. Moreover, IL-10 expression was correlated with thyroid tumor size and malignancy [57]. Interestingly, B cells from TC patients produced higher levels of IL-10 compared to healthy controls [58].

IL-12, a proinflammatory cytokine with antitumor activity [59], was effective against ATC in a mouse xenograft model [60], and its administration inhibited tumor growth and prolonged survival by activating both CD8^+^ T and natural killer (NK) cells in a genetically engineered *BRAF^V600E^* mouse model [61].

IL-17A is produced mainly by human CD4^+^ Th17 cells and to a lesser extent by CD8^+^ T cells (Tc17 cells), and was associated with both protumor or antitumor responses [62,63]. The serum concentration of IL-17 was increased in TC patients compared to thyroid adenoma [64] and correlated with the percent of circulating Th17 cells [62]. *IL17RA* polymorphisms negatively correlated with the development of PTC [65]. The serum levels of IL-17 and the percentage of Th17, Tc17, and T regulatory cells (Tregs) were increased in patients with DTCs compared to healthy controls [66]. IL-17 and IL-23 expression in DTCs and MTCs were increased compared to thyroid adenoma, and IL-17 expression was associated with recurrence and mortality [67].

IL-21 is highly expressed by both Tfh and Th9 cells [68]. In a large cohort of a Chinese population, IL-21 polymorphism was associated with an increased risk of TC development [69].

IL-24 was induced by RET/PTC3 expression in the thyrocytes of RET/PTC3 transgenic mice, and it was involved in autocrine growth/survival of RET/PTC3-expressing thyroid cells supporting its role in early cellular transformation. Its expression decreased in poorly differentiated mouse tumors, paralleling the loss of RET/PTC3 expression along with tumor progression, thus supporting a role for IL-24 as a tumor suppressor factor [70].

In a murine model of transgenic BRAF, mouse thyrocytes and TAMs produced TGF-β, which was responsible for the acquisition of EMT features and the invasiveness of TC cells [71]. In human PTCs, TGF-β overexpression correlated with tumor invasiveness, regardless of BRAF mutations [72]. The expression of TGF-β in PTC correlated with CD68^+^ cell infiltration in PTC [73] compared to benign thyroid nodules [74]. These findings led to suggest that targeting *transforming growth factor beta* β1 (TGF-β1) could inhibit ATC tumorigenesis [75].

All three classes of interferons (IFNs), type I (IFN-α/β), type II (IFN-γ), and type III (IFN-λ/s), induce the apoptosis of tumor cells and modulate cancer immunosurveillance [76]. In TC, type I and type II IFNs induced the expression of Major Histocompatibility Complex (MHC)-I molecules on human TC cell lines, thus limiting TC immunoevasion and potentiating TC susceptibility to immune destruction [77]. IFN-γ inhibited the migration of PTC cells in vitro and induced EMT [78]. Moreover, IFN-γ inhibited CXCL8 secretion from *BRAF^V600E^* mutated thyroid cell lines [79].

## 4. Chemokines

Chemokines are functionally related small molecules with chemoattractant and cytokine-like functions that also modulate angiogenesis and lymphangiogenesis [80]. Chemokines can be produced by TC cells, following the activation of the MAPK pathways by the RET/PTC, RAS, and BRAF oncogenic drivers [81,82]. Thyroid cells produce a wide spectrum of CXC chemokines (i.e., CXCL1, CXCL8, CXCL9, CXCL10, and CXCL11) in basal conditions and/or under the influence of specific stimuli [83,84]. In thyroid tissue, recruited T helper 1 (Th1) lymphocytes can enhance IFN-γ and tumor necrosis factor α (TNF-α) production, which in turn stimulate the chemokine secretion from the thyroid cells, therefore creating an amplification feedback loop, initiating and perpetuating the autoimmune process. PTC and ATC cell lines release CXCL8 in basal conditions as well as under inflammatory stimuli, such as IL-1 and TNF-α [40,85]. The exogenous induction of RET/PTC1 oncogene in primary normal human thyrocytes induced the expression of *CCL2, CCL20,* and *CXCL8* genes. *CCL20* and *CXCL8* were up-regulated in clinical samples of PTC [82]. PTCs displayed the highest expression of *CCL20* and *CXCL8* compared to normal tissues and thyroiditis, regardless of the genetic lesion beard by the tumor [86]. In a model of orthotopic TC xenograft in nude mice, CXCL8 was involved in tumor growth and progression [87]. Also, the tumor-infiltrating immune cells can be a source of chemokines. Indeed, mast cell-derived CXCL8 in vitro induced EMT features and stemness in human TC cells [48]. TAMs purified from PTC patients released CXCL8 [88].

PTC and ATC cell lines produce CXCL1/GRO-α and CXCL10/IP10 in basal conditions and under inflammatory stimuli [81,83,89]. Moreover, mast cell-derived CXCL1/GRO-α (growth-regulated oncogene α) and CXCL10/IP10 increased TC cell proliferation through the engagement of CXCR2 and CXCR3 on TC cells [47,81]. CXCL12/SDF-1 and its receptor CXCR7 have been found in PTC [90]. The CXCL12/SDF-1-CXCR7 axis promoted in vitro TC cell line proliferation and invasion [91]. Also, CCL20 promoted TC cell invasion and migration in vitro [92]. Two splice variants of CXCR3 (A and B), the receptor on follicular thyroid cells activated by several chemokines (CXCL4, CXCL9, CXCL10, and CXCL11), have been found [93]. A progressive increase in CXCR3A expression over the CXCR2B isoform was found from benign tumors to PTC.

We have found that the oncolytic adenovirus *dl922-947* reduced CXCL8 and CCL2 production by ATC cell lines. *dl922-947* treatment impaired angiogenesis and favored the switch of tumor macrophages toward an M1 phenotype. These results indicate that *dl922-947* treatment, along with its role in inducing TC cell death, has an impact on the ATC immune microenvironment.

## 5. Angiogenic and Lymphangiogenic Factors

**Figure 1:**
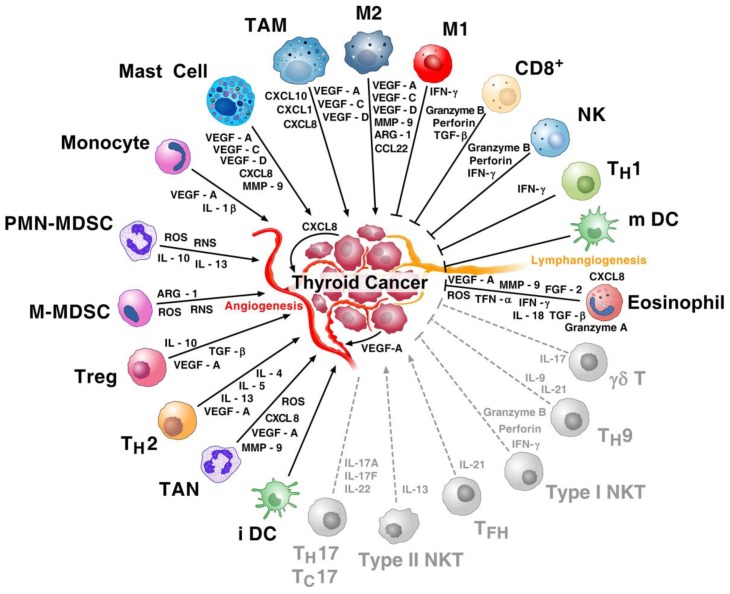
Hypothetical scheme of immune contexture of thyroid cancer (TC). The immune network in thyroid cancer is a complex and dynamic system characterized by multiple interactions between tumor cells and nearly all immune cells. Tumor-associated macrophages (TAM), M2 macrophages, tumor-associated mast cells, monocytes, polymorphonuclear-myeloid-derived suppressor cells (PMN-MDSCs), monocyte-derived suppressor cells (M-MDSCs), T regulatory cells (Treg) and T helper 2 (Th2) cells, tumor-associated neutrophils (TAN), and immature DCs (iDCs) and their mediators play protumorigenic roles in thyroid cancer. M1 macrophages, cytotoxic CD8^+^ T cells, natural killer (NK) cells, Th1 cells, mature DCs (mDCs), and their mediators play an antitumorigenic role. There is increasing evidence that eosinophils play an antitumorigenic role in different cancers [102,108,109]. VEGF-A and CXCL8 produced by thyroid cancer cells activate tumor angiogenesis. Mast cells and macrophages are major producers of lymphangiogenic factors (VEGF-C and VEGF-D). The antitumorigenic role of γδ T cells, Th9 cells, and type I natural killer T (NKT) cells (grey and dashed lines) have been demonstrated in several other human cancers. The protumorigenic role of Tfh cells and of type II NKT cells has been shown in several other human tumors (grey and dashed lines). Protumor or antitumor activities of Th17 and Tc17 cells are context-dependent (grey and dashed line). Modified with permission from Galdiero et al. [110].

The formation of blood and lymphatic vessels is a complex process, requiring a finely tuned balance between stimulatory and inhibitory signals such as vascular endothelial growth factors (VEGFs), angiopoietins (ANGPTs), chemokines, and many others [94,95,96]. Several TC-infiltrating immune cells can impact tumor angiogenesis and lymphangiogenesis. Figure 1 shows that a plethora of cytokines, chemokines, angiogenic factors, and lymphangiogenic factors derived from immune cells enrich the complexity of the inflammatory tumor microenvironment in TC. For instance, mast cells (MCs) are a major source of proangiogenic (VEGF-A and VEGF-B) and lymphangiogenic (VEGF-C and VEGF-D) factors [97]. Thyroid MCs modulated angiogenesis through the release of CXCL8 [48]. Human macrophages are also a major source of both angiogenic and lymphangiogenic factors [98], as well as of several proangiogenic enzymes such as matrix metallopeptidase 9 (MMP-9), cyclooxygenase -2 (Cox-2), andinducible nitric oxide synthase (iNOS) [44]. Tumor-associated NK cells produce VEGF-A and CXCL8 [99]. Tumor-associated DCs release VEGF-A, CXCL8, and osteopontin [100]. Human eosinophils promote angiogenesis [101,102], and eosinophilia can be observed in ATC patients [103]. Tregs play a role in maintaining tolerance in TC and drive tumor angiogenesis through the release of VEGF-A [104]. Myeloid-derived suppressor cells (MDSCs) promote tumor angiogenesis through the release of VEGF-A and MMP-9 [105]. Human Th2 cells and neutrophils are sources of angiogenic factors [94,106,107].

## 6. The Immune Landscape in TC

The tumor microenvironment (i.e., immune cells, fibroblasts, blood and lymphatic vessels, endothelial cell progenitors, and extracellular matrix components (ECM)) plays a central role in tumor initiation and progression. The normal tissue microenvironment can suppress malignancy, while certain pathogenetic tissue features can induce tumor progression [111,112].

### 6.1. Tumor-Associated Macrophages (TAM)

Macrophages are key components of the tumor microenvironment and are highly plastic cells [113,114,115]. Under the influence of IFN-γ, macrophages undergo M1 polarization, which is characterized by an immunostimulatory phenotype. In contrast, IL-4 or IL-13 induce the M2 phenotype, which promotes tumor angiogenesis and suppresses immune responses [44]. This model distinguishing between classically polarized antitumor M1 and alternatively polarized M2 subtypes incompletely accounts for the extraordinary phenotypic diversity of macrophages in vivo [116,117]. High-resolution analysis of TAMCs led to the identification of 17 major phenotypes of human macrophages [116].

In PTCs, TAMs correlated with lymph node metastasis [88], larger tumor size [118], and reduced survival. In PDTC, TAM density correlated with capsular invasion and extrathyroid extension [32]. TAMs represent more than 50% of immune cells in ATCs, forming a “microglia-like” in close contact with cancer cells [31]. In the diffuse sclerosing variant of PTC, M2-like macrophages can be found in lymphatic emboli and correlated with tumor cell lymphatic invasion [119].

Macrophages are present in the immune landscape of PTC, especially in the *BRAF^V600E^*^+^ group [120]. The histologic grading of CD68^+^ TAM increased in more aggressive thyroid cancers (i.e., PDTC and ATC) compared to PTC [121]. In a murine model of transgenic *BRAF^V600E^*-induced thyroid carcinogenesis, tumors displayed a high TAM infiltration due to the increased expression of *Csf-1r* and *Ccr2* by tumor cells. In this model, TAMs displayed an M2-like phenotype. In addition, Csf-1 ^−/−^ BRAF transgenic mice displayed a reduction in tumor growth [45]. TAMs purified from human PTC displayed a higher expression of IL-10 and CD206 compared to peripheral blood monocytes [122], and promoted the invasiveness of TC cell lines in vitro through the production of CXCL8 [88]. TAMs are present in ATC to variable degrees, ranging from 22% to 95% [31,32,123]. ATCs and to a lesser extent PDTCs have an extensive infiltration of macrophages [31,32], which make an interconnected network that envelops the tumor cells throughout the cancer specimen. A total of 68 genes that were overexpressed in M2 macrophages were examined in PDTCs and ATCs, and M2 signatures clearly differentiated the two TCs [34]. The role of macrophages in the formation of lung metastasis has been evaluated in an experimental model of ATC [124]. Macrophage depletion in mice injected with clodronate reduced the lung metastasis of ATC cell lines. Metastasis-associated lung adenocarcinoma transcript 1 (MALAT1) is a lung non-coding RNA that is up-regulated in several cancers, including TC [125]. MALAT1 is up-regulated in TC tissues and cells, and induced the expression of basic fibroblast growth factor (FGF2) and IL-10 in TAM, which promoted FTC133 proliferation and angiogenesis [126].

Soluble factors released by TC cell lines (TPC1, BC-PAP, and FTC133) induced an inflammatory phenotype of peripheral blood monocytes [127]. Zhang et al. examined the protumorigenic role of testosterone using the *Thrb^PV/PV^* transgenic mouse model, which mimics human FTC development. They found that testosterone reduced the expression of the immune regulatory genes *Glipr1* and *Sfrp1*, the M1 macrophage, and CD8^+^ T cell infiltration in thyroid samples. These immunosuppressive events resulted in TC progression in *Thrb^PV/PV^* mice [128]. These interesting findings were corroborated by the analysis of the database of the National and Cancer Institute’s Surveillance Epidemiology End Results (NIH, Bethesda, USA), which found that men had a higher rate of large primary or locally advanced FTC than women. Furthermore, there was higher FTC-associated mortality in men than in women in the 40-to-60-year age group.

High-dimensional analysis, particularly single-cell RNA-seq, will be necessary in order to better characterize the role of macrophages in thyroid tumorigenesis.

### 6.2. Dendritic Cells (DCs)

Tumor-infiltrating dendritic cells (DCs) display an immature phenotype, with impaired antigen presentation ability [129]. S100^+^ (mature and immature), CD1a^+^ (immature), and CD83^+^ (mature) DCs are increased in human PTC compared to normal thyroid tissue [130]. Conditioned media from the primary cultures of PTC induced the chemotaxis of peripheral blood monocyte-derived DCs. Hepatocyte growth factor (HGF) enhanced this chemotactic activity through the engagement of the receptor Met on TC cells [131]. PTCs expressed the DC chemotactic molecule macrophage inflammatory protein 1α (MIP-1α), and DCs expressed CCR6, thus suggesting a role for TC cells in recruiting DCs [132]. PTC displayed a higher CD1a^+^ DC infiltration compared to FTC and adenomas [132]. In contrast, a reduced or absent DC infiltration in PDTC and ATC was described in comparison to DTC [133]. DCs are present in the immune landscape of PTC, especially in the BRAF^V600E+^group [120].

### 6.3. Tumor-Associated Mast Cells (TAMCs)

Mast cells are ubiquitous in nearly all tissue and in close proximity to epithelia, fibroblasts, blood vessels, lymphatic vessels, and nerves [95,134]. Mast cells can play a role in angiogenesis [134], lymphangiogenesis [97], and tumor initiation and progression [110,135,136]. Mast cells are present in the microenvironment of several solid [47,137,138,139,140,141,142,143] and hematologic tumors [144]). The contribution of mast cells in cancer varies according to the stages of tumorigenesis and to their microlocalization [142].

Melillo et al. were the first to investigate the contribution of mast cells in thyroid cancer. Mast cell density was very low in normal thyroid tissue, whereas in PTC samples, there was a mast cell infiltration that correlated with tumor extrathyroid extension [47]. These findings were extended to a limited number of PDTCs and ATCs [48]. The presence of mast cells in the immune landscape of PTC has been recently confirmed [120].

A higher mast cell density was also found in FTC compared with adenomas and correlated with extracapsular extension [145]. In vitro studies demonstrated that TC cell line conditioned media induced mast cell chemotaxis through the release of VEGF-A [47], which activated the VEGFRs on human mast cells [97,146]. Moreover, TC cells activated mast cells to release cytokines (IL-6, TNF-α, granulocyte-macrophage colony-stimulating factor—GM-CSF) and chemokines (CXCL10/IP10 and CXCL1/Gro-α). In turn, mast cells promoted TC cell proliferation through CXCL1/GROα and CXCL10/IP10. In an in vivo model of a TC xenograft, mast cells were recruited at the tumor site and accelerated tumor growth, enhancing tumor vascularization and cell proliferation of the xenograft [47]. These results indicate that mast cells are present in human TCs and play a protumorigenic role in TC.

Epithelial-to-mesenchymal transition (EMT) is important in tumor progression, and is in part responsible for the acquisition of the invasive properties of cancer cells [147]. A mast cell-conditioned medium induced the EMT of human TC cell lines mainly through the release of CXCL8 [48]. Interestingly, the blockade of CXCL8 receptors (i.e., CXCR1 and CXCR2) with blocking antibodies markedly reduced the sphere-forming ability of TC cells [48]. Collectively, these findings support the hypothesis that mast cell-derived CXCL8 favors the acquisition of stem-like features of TC cells.

Recent evidence indicates that mast cells, similar to macrophages [116,148] and neutrophils [107], comprise several subsets of cells [149,150]. Single-cell RNA-seq analysis will be necessary to better understand the role(s) of mast cells subsets in TC development and the formation of metastases.

### 6.4. Tumor-Associated Neutrophils (TANs)

Neutrophils participate in the early phases of inflammation and resistance against extracellular pathogens [107,151], and play a role in cancer initiation and growth [152]. In humans, the ratio between peripheral blood neutrophil and lymphocyte count (neutrophil-to-lymphocyte ratio—NLR) has been proposed as an index of systemic inflammation, and has been found to be associated with tumor development [153]. A higher NLR was associated with a larger tumor size and higher risk of recurrence in TC patients, but failed to distinguish patients with benign or malignant nodules [154]. In contrast, an increased NLR has been found in TC compared with benign lesions and healthy controls [155]. No correlation was found with patient disease-free survival or risk of occult metastasis [156]. In a large cohort of PTC, PDTC, and ATC, NLR was increased in ATC and to a lesser extent in PDTC [157]. In a meta-analysis based on seven prospective cohorts comprising 7349 patients, no difference in NLR was found between DTC and patients with benign nodules [158]. Preoperative NLR was not associated with clinicopathological characteristics in PTC patients [159,160]. By contrast, other studies reported that the preoperative NRL was correlated with the size and lymph node metastasis of PTC patients [161,162,163,164]. In conclusion, the diagnostic and prognostic significance of NLR in different types of TC remains uncertain.

Maria Rosaria Galdiero et al. elegantly investigated the potential involvement of neutrophils in human TC [165]. Conditioned media from TC cell lines TPC1 and 8505 (TC-CM) promoted human neutrophils chemotaxis and survival. Neutrophil chemotaxis was mediated at least in part by CXCL8, and survival was mediated by GM-CSF. TC-CM induced morphological changes and the activation of neutrophils (i.e., CD11b and CD66 overexpression and CD62L shedding). Moreover, TC-CM induced the production of ROS and angiogenic mediators (i.e., VEGF-A and CXCL8). Importantly, the density of tumor-infiltrating neutrophils correlated with TC size. This study was the first to reveal the possible involvement of neutrophils in human TC.

Due to their relatively low transcriptional activity and short lifespan [166], neutrophils are generally believed to be terminally differentiated homogeneous cells once they leave the bone marrow. This view is rapidly changing, and various subsets of neutrophils that are mostly defined by surface markers [167] and density [168] have been described. Studies in murine models of cancer have identified two functionally antagonistic populations of neutrophils, which are referred to as N1 and N2 to mirror the nomenclature of M1 and M2 macrophages with similar activity [152]. The lack of definitive cell markers of human N1 and N2 subsets has been so far a major obstacle to establish their role in TC.

### 6.5. Myeloid-Derived Suppressor Cells (MDSCs)

Myeloid-derived suppressor cells (MDSCs) are largely immature myeloid cells that are characterized by a state of activation and display potent immune suppressive activity [169]. Two major subsets of MDSCs have been identified so far: monocytic (M-MDSCs) and polymorphonuclear (PMN-MDSCs) [170]. M-MDSCs share phenotypic and morphologic features with monocytes, whereas PMN-MDSCs are similar to neutrophils. MDSCs have been implicated in tumor immune responses, tumor initiation and progression, angiogenesis, and the formation of pre-metastatic niches [169,170]. MDSCs inhibit anti-cancer immune responses by releasing cytokines (IL-1 and TGF-β), ROS, and reactive nitrogen species (RNS) that condition the tumor microenvironment and by stimulating Foxp3^+^ Treg cells and M2 tumor-associated macrophages (TAM) [171]. Moreover, MDSCs promote angiogenesis and condition the pre-metastatic niches [170]. M-MDSCs and PMN-MDSCs differentiate from normal progenitors of monocytes and neutrophils, respectively [172]. Recently, PMN-MDSCs were distinguished from human neutrophils by the expression of the lectin-type oxidized LDL receptor 1 (LOX-1). LOX-1^+^ neutrophils were potent suppressor of T cells, while LOX-1^−^ neutrophils, presumably classical neutrophils, were not [173,174].

Peripheral blood MDSC levels were increased in patients with ATC compared to healthy controls and correlated with the serum level of IL-10, suggesting a correlation between MDSCs and systemic immunosuppression [175]. MDSCs are attracted to tumor sites in response to various cytokines (CCL2, CCL5, and CSF1 for M-MDSCs, and CXCL1, CXCL5, CXCL6, CXCL8, and CXCL12 for PMN-MDSCs) [176]. In tumors, the incoming cells dive into a fairly hostile microenvironment characterized by hypoxia, low pH, and high concentrations of cytokines, lactate, adenosine, and oxidative agents (ROS, NO) [176,177]. These conditions heavily affect MDSC survival, functions, and differentiation. It is generally thought that PMN-MDSCs in tissues are short-living cells, and that monocytic cells in the tumor microenvironment most likely represent bona fide M-MDSCs. In a wide cohort of TC patients (253 PTCs and 13 FTCs), the intratumoral MDSCs count, which were identified as CD11b^+^ and CD33^+^ cells, did not correlate with patient clinicopathological features [178]. Two studies did not find increased peripheral blood MDSCs, which were defined as CD11b^+^ CD33^+^ cells, in PTC patients compared to control, but showed accumulation in patients with ATC [175,179]. More recently, circulating CD11b^+^ HLA-DR^low^ (presumably PMN-MDSCs), but not CD33^+^ HLA-DR^low^ (presumably M-MDSCs), were increased in DTC, mostly PTC, compared to benign nodules [180]. The lack of definite cell markers for human MDSC subsets has been so far a major obstacle to establish their clinical significance in TC patients. Single-cell RNA-seq will be needed to identify subsets of M-MDSCs and PMN-MDSCs in different types of human TCs.

A mouse model *Hras^G12V^/Pten/TPO-Cre* developed multifocal FTC, which displayed many of the classical hallmarks of high-grade human FTC and PDTC, including an extrathyroidal extension and lung metastasis [181]. These tumors were heavily infiltrated by macrophages, MDSCs, and double-positive CD4^+^ CD25^+^ T cells (Treg cells), and contained arginase-1^+^ cells. These findings are suggestive of an immunosuppressive tumor microenvironment (TME) of TC.

### 6.6. Natural Killer (NK) Cells

Natural killer (NK) cells are a family of innate immune cells that play crucial roles in protective immunity against tumors and viral infections [182]. Subtypes of human NK cells have been identified on the basis of the relative surface expression of CD16 and CD56. CD56^dim^ CD16^+^ NK cells display a higher cytotoxic activity, whereas CD56^bright^ CD16^−/low^ NK cells are more efficient in cytokine production [183]. Tumor-infiltrating NK cells were increased in PTCs compared to goiters and healthy thyroids, whereas no differences were found in peripheral blood NK cells [184,185]. In PTC patients, NK cell infiltration negatively correlated with disease stage [186]. An increased infiltration of the immunoregulatory subset of NK cells CD56^bright^ was found in PTC samples compared to MNG. CD56^bright^ NK cells inversely correlated with the disease stage, whereas cytotoxic NK cells positively correlated with the disease stage in PTC patients. These results indicate that the TC microenvironment modulates the phenotype of NK cells [187].

NK cells mediated the lysis of ATC cell lines through the expression of the activating receptor NKG2D on NK cells and its ligands UL16 binding proteins (ULBP2/5/6) on ATC cells [188]. ATC cell lines and TC derived from human fine-needle aspiration [189] samples from ATC patients expressed ULB2/5/6, whereas non-malignant thyroid tissue did not. ATC cell lines induced NK cell migration through the activation of the CXCL10–CXCR3 axis. NK cells from FNA and the peripheral blood of ATC patients displayed a suppressed phenotype, which was characterized by a lower percentage of CD56^dim^ cells and CXCR3^+^ cells and a reduced NKG2D expression, compared to peripheral blood NK cells. PGE_2_ produced by thyroid cancer cells [190] can be responsible for ATC-mediated NK cell suppression [188]. A recent study found a decrease frequency of peripheral blood cytotoxic NK cells (i.e., CD56^lo^ CD16^hi^) in ATC patients compared to controls [191]. By contrast, NK cells CD56^hi^ CD16^lo/hi^ (cytokine producing) were increased in ATC compared to non-ATC patients and healthy controls.

In a knock-in mouse model, BRAF oncogene was expressed under the control of the thyroid peroxidase (TPO) promoter (LSL-BRAF^V600E^/TPO Cre mice). IL-12 gene therapy and recombinant IL-12 reduced tumor growth, restored the follicular architecture of the gland, and improved the survival of tumor-bearing mice. Interestingly, IL-12 improved the cytotoxicity of CD8^+^ T cells and NK cells, and increased the infiltration of M1 macrophages within the tumors [61]. NK cells inhibited the growth of metastasis in an in vivo mouse model of ATC pulmonary metastasis [192].

### 6.7. Natural Killer T Cells (NKT)

NKT cells are a heterogeneous lymphoid population that recognizes the lipid antigens presented by CD1d and plays an important role in tumor immunosurveillance [193]. Two NKT subsets have been identified: type I induce the lysis of tumor cells directly via a perforin/granzyme B-mediated mechanism or indirectly via the activation of NK cells and DCs; type II show immunosuppressive activity through the production of IL-13 [194]. NKT subsets have not been characterized in TC.

### 6.8. γδ T Cells

γδ T lymphocytes, expressing a γδ T cell receptor (TCR), are not MHC-restricted and do not recognize peptide antigens [195]. The contribution of γδ T cells in tumor immunosurveillance is controversial. Zitvogel et al. demonstrated that IL-17-producing γδ T cells play a prominent role in chemotherapy-induced anti-cancer immune responses [196]. The role of tumor-infiltrating γδ T cells in TC is still unknown.

### 6.9. Innate Lymphoid Cells (ILCs)

Innate lymphoid cells (ILCs) lack TCR, but functionally resemble effector T cells [197]. ILCs include three subsets of cytokine-producing helper cells: group 1 ILCs produce IFN-γ and include conventional NK cells; group 2 produces Th2-type cytokines (e.g., IL-4, IL-5, and IL-13); group 3 ILCs comprises several distinct cell subsets [198]. ILCs have a critical role in the development of lymphoid structures, the maintenance of immune homeostasis, tissue remodeling, and the maintenance of epithelial integrity [199]. ILCs have also been implicated in the control and suppression of tumors [200]. There is evidence that the tumor microenvironment dictates the fate of the tumor-suppressive functions of ILC2. Moreover, ILC2 can modulate T cell-to-MDSC balance in cancer [201]. Studies are required to understand the role of ILC subsets in different TCs.

### 6.10. CD8^+^ Cytotoxic T Cells

CD8^+^ cytotoxic T lymphocytes (CTLs) recognize and attack tumor cells expressing tumor antigens [202]. In a wide immunohistochemical characterization of the immune network in patients with chronic lymphocytic thyroiditis concurrent with DTC, a high CD8^+^ T lymphocyte infiltration was associated with improved disease-free survival [178]. In a study conducted in a wide cohort of DTC patients, including papillary and follicular subtypes, immunohistochemical analysis of tumor samples revealed that the combined enrichment of CD8^+^ cells and Cox-2 overexpression correlated with the highest risk of disease relapse. In the majority of the tumor samples analyzed (68%), CD8^+^ cells were granzyme B negative, reflecting a state of anergy [203].

A low intratumoral CD8^+^/Foxp3^+^ ratio was found in human BRAF^V600E^ PTC, which was associated with an increased expression of the immunosuppressive molecules arginase-1, indoleamine 2,3-dioxygenase (IDO), and programmed death-ligand 1 (PD-L1). The latter findings suggest a BRAF-driven tumor-promoting microenvironment [204]. A recent study evaluated the CXCR5^+^ CD8^+^ T cell subset in peripheral blood, tumor-draining lymph nodes (TDLNs), and tumors from TC patients [205]. Although CXCR5^+^ CD8^+^ T cells expressed higher PD-1, T-cell immunoglobulin and mucin-domain containing-3 (TIM-3), and Cytotoxic T-Lymphocyte Antigen 4 (CTLA-4) markers than CXCR5^−^ CD8^+^ T cells, these cells displayed a higher expression of cytotoxic molecules (e.g., granzymes and perforin).

### 6.11. CD4^+^ Cells

The role of different subsets of CD4^+^ T cells in tumor immunity remains underappreciated. Th1-mediated immunity is generally considered as antitumoral [206], while polarized Th2 and/or Treg activity is believed to be protumorigenic [207]. This simplistic view is complicated by the plasticity of Th differentiation, which can be extensively modulated by the tumor microenvironment [115,208]. In TC, the extent of tumor-infiltrating CD4^+^ cells does not appear to predict patient outcome [203]. No differences were found between PTC and MNG patients, with respect to tissue or peripheral blood CD4^+^ cell frequencies [53]. Interestingly, a double negative CD4^−^ CD8^−^ lymphocyte population was the dominant cell type in PTC, and was more abundant in PTC than in thyroid autoimmunity and ex vivo released IFN-γ and IL-17 [209].

### 6.12. Treg Cells

Tregs shut down antitumor immune response via the production of IL-10, the expression of immunosuppressive molecules (e.g., CTLA-4 and PD-1), and the stimulation of angiogenesis [210]. Moreover, increased PD-1^+^ T and Treg cells in metastatic lymph nodes correlated with a more aggressive TC [211]. Foxp3^+^ Tregs and VEGF were found in PTC samples, and Treg infiltration correlated with disease stage and lymph node metastasis [212]. A higher Treg density was also observed in PTC samples compared with nodular goiter, and positively correlated to the stage of the disease [186]. Accordingly, a high infiltration of Foxp3^+^ Treg cells was associated with aggressive features of PTC [213]. Moreover, increased Foxp3^+^ and reduced CD3^+^ tumor-infiltrating lymphocytes correlated with IDO1 expression. Similarly to PD-L1, IDO1 is overexpressed in different tumors, and is associated with the activation of Foxp3^+^ Tregs and the down-regulation of cytotoxic cellular immunity in the tumor microenvironment [214].

A higher percentage of Foxp3^+^ T cells and Inducible T-cell COStimulator (ICOS) ^+^ Treg cells were found in tissues, but not in the peripheral blood of PTC patients with MNG compared to MNG alone [53]. In PTC plus MNG, tissue ICOS^+^ Foxp3^+^ T cells were increased in advanced stages and metastatic tumors. Tissue ICOS^+^ Foxp3^+^ T cell numbers correlated with tissue plasmocytoid DCs, which favor an immunosuppressive microenvironment [53].

### 6.13. IL-17^+^ Cells

CD4^+^ IL-17^+^ T cells (Th17) cells can exert protumor or antitumor functions, depending on the tissue microenvironment [215]. Only one study examined the prevalence and distribution of Th17 cells in TC samples. In peripheral blood and tissue samples of PTC patients, increased Th17 levels were found compared to healthy controls, whereas the percentage of CD8^+^IL-17^+^ T cells (Tc17) in the peripheral blood was reduced. The frequency of peripheral blood Th17 cells was positively correlated with the IL-17 serum level, while no correlation between the serum level of IL-17 and Tc17 cells was found. Peripheral blood Th17 cells inversely correlated with tumor size [62].

### 6.14. T Follicular Helper Cells (Tfh)

T follicular helper cells (Tfh) were discovered based on their expression of the essential transcription factor BCL6 [42,216]. This led to the recognition of Tfh cells as an independent CD4^+^ subset specialized in helping B cells in lymph nodes [42,217]. No single marker or combination of markers reliably identifies Tfh cells as a discrete immune cell population. In fact, Tfh are part of the CD4^+^ T cell differentiation spectrum. Several surface molecules (CCR5, PD-1, BCL6, BTLA4, and ICOS) vary in expression and represent plastic features of Tfh heterogeneity. A fraction of CD4^+^ T cells in human blood express CXCR5 or PD-1 and are called circulating Tfh (cTfh) cells. The latter cells are closely related to tissue Tfh cells. Human cTfh are heterogeneous [42], and are divided into three major functional subtypes (Tfh1, Tfh2, and Tfh17) [218]. IL-21 is a B-cell helper cytokine produced by Tfh cells [219].

Overreactive Tfh cells have been reported in several human systemic autoimmune diseases [220]. An increased frequency of cTfh cells has been reported in patients with autoimmune thyroid disease, and Tfh cells were also detected in the thyroid tissue of Hashimoto’s thyroiditis patients [221]. CXCR5^+^ CD4^+^ T cells were increased in the thyroid tissue of patients with Graves’ disease compared to control subjects [222]. Furthermore, CD4^+^ IL-21R^+^ T cells and CD19^+^ IL-21R^+^ B cells were also observed in Graves’ disease tissues.

Recent studies suggested that Tfh cell infiltrates may influence the growth and survival of certain tumors [223,224]. To our knowledge, the characterization of different subtypes of circulating and intratumor Tfh in different types of TC has not been reported yet. Future studies should investigate the presence and functions of Tfh subsets in different types of TC.

### 6.15. Th9 Cells

**Figure 2:**
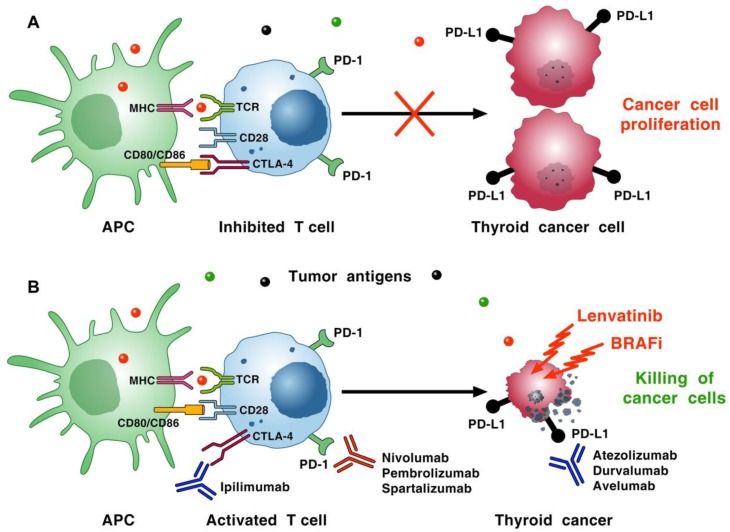
Schematic representation of the rationale developing combinatorial therapies of advanced thyroid cancer involving immune checkpoint inhibitors (monoclonal antibodies (mAbs) anti-cytotoxic T lymphocyte antigen 4 (anti-CTLA-4), anti-programmed cell death protein-1 (anti-PD-1), or anti-programmed cell death ligand-1 (anti-PD-L1)), BRAF inhibitors (BRAFi), multi-targeted tyrosine kinase inhibitors (TKIs) (e.g., lenvatinib), chemotherapies, or radiotherapies. (**A**) Cancer cells release neoantigens (dots of different colors) that are captured by antigen-presenting cells (APCs). These cells present peptides in the context of MHC I molecule/T cell receptor (TCR) on the surface of CD8^+^ cytotoxic T cells. APCs can also present peptides bound to MHC II molecules on CD4^+^ T helper cells. T-cell activation requires costimulatory signals transmitted via CD28, which is activated by binding to CD80 and/or CD86 on APCs. Tumor cells up-regulate CTLA-4 on T cells, which competes with CD28 for binding to CD80/CD86 on APCs. The interaction of CTLA-4 with CD80/CD86 results in inhibitory signaling in T cells, which favors thyroid cancer cell proliferation. The immunosuppressive activity of CTLA-4 is mediated by the down-regulation of Th cells and the enhancement of Treg cells. Moreover, tumor cells express high levels of PD-L1 and/or PD-L2, which binds to PD-1 on T cells, resulting in inhibitory signals that decrease cytotoxicity and lead to T-cell exhaustion. (**B**) mAbs blocking CTLA-4 (e.g., ipilimumab, tremelimumab), PD-1 (nivolumab, pembrolizumab, spartalizumab), or PD-L1 (avelumab, atezolizumab, durvalumab) inhibit the interactions of CTLA-4/CD80/86 and PD-1/PD-L1, respectively, and activate T-cell cytotoxicity. BRAF inhibitors (BRAFi), TKIs (e.g., lenvatinib), chemotherapies, and radiotherapies can induce thyroid cancer cell death, increasing the release of tumor neoantigens in the tumor microenvironment. Combining an anti-PD-L1 antibody with BRAFi [228,229] or with lenvatinib [229] improved survival and tumor immunity in a immunocompetent murine model of ATC. Several combination strategies involving immune checkpoint inhibitors (ICIs) are under evaluation in patients with advanced TC (see Table 1 and Table 2).

IL-9-producing CD4^+^ helper T cells (Th9 cells) are a subset of CD4^+^ helper T cells with proinflammatory functions and anti-cancer properties in vivo [225]. The release of IL-9 has been proposed to account for anti-cancer efficacy. Moreover, Th9 cells release IL-21, which promotes the production of IFN-γ and tumor elimination by CD8^+^ T cells and NK cells [226]. The Th9 antitumor efficacy has been attributed to the production of IL-9 [227]. Studies investigating the relevance of Th9 cells in thyroid oncogenesis are urgently needed. Figure 2 schematically illustrates a hypothetical immune landscape of TC.

## 7. Immune Checkpoint Inhibitors in TC

Immune checkpoints physiologically prevent excessive immune responses and the development of autoimmunity [230,231,232]. Monoclonal antibodies (mAbs) targeting immune checkpoints (immune checkpoint inhibitors: ICIs) have revolutionized the treatment of malignancies characterized by DNA microsatellite instability [233]. Two major classes of ICIs have clinically emerged: those targeting cytotoxic T lymphocyte antigen 4 (CTLA-4) (i.e., ipilimumab and tremelimumab), and those targeting programmed cell death protein-1 (PD-1) (i.e., nivolumab, pembrolizumab, spartalizumab) or its ligand, programmed cell death ligand-1 (PD-L1) (i.e., avelumab, atezolizumab, and durvalumab). CTLA-4 and PD-1 regulate different stages of the immune response. For instance, CTLA-4 modulates immune response primarily in draining lymph nodes, whereas the primary site of action of PD-1 and its ligands (PD-L1/PD-L2) is in the tumor microenvironment (TME). PD-1 expressed on activated T cells interact with PD-L1 or PD-L2 expressed on the surface of cancer cells or tumor-infiltrating immune cells, thus inhibiting the cytotoxic action of T cells [234]. The up-regulation of PD-L1 by tumor cells leads to increased T-cell exhaustion, and is thought to be a means of cancer cell immune evasion [235]. When T cells are primed by exposure to antigens presented by APCs in draining lymph nodes, they become activated and overexpress CTLA-4 on their surfaces, which competes with CD28 for binding to CD80 or CD86 on the surface of APCs. This competition between CTLA-4 and CD28 attenuates the early activation of CD4^+^ and CD8^+^ T cells, and improves the immunosuppressive functions of Treg cells [236]. Figure 2A schematically illustrates the two interactions between CTLA-4 on T lymphocytes and CD80/CD86 on APCs and between PD-1 on T cells and tumor cells expressing PD-L1 or PD-L2. Both interactions result in inhibitory signals that lead to T-cell exhaustion, decreased T-cell cytotoxicity, and cancer cell proliferation.

The PD-1/PD-L1 pathway is expressed in ATC and DTC [237,238]. *BRAF*^V600E^ cells express higher expression of PD-L1 compared to *BRAF*^WT^ cells [228]. The low rate of mutations—and therefore, of neoantigens in DTC—might suggest that this would be a poor target for monotherapy with ICIs [239]. In fact, the results of a preliminary trial of the PD-1 inhibitor pembrolizumab in patients with PD-L1^+^ PTC or FTC confirmed that hypothesis [240]. More recently, higher numbers of mutation load and genetic alterations have been identified in poorly DTCs and ATC than in DTCs [34]. To increase the response rate of ICIs, a major focus is to find combinations that act synergistically. For example, combination strategies that target multiple aspects of the immune response (e.g., PD-1/PD-L1 and CTLA-4 pathways) or tumor cells themselves (e.g., tyrosine kinase inhibitors, BRAF inhibitors, radiotherapy) could provide benefits for patients with advanced thyroid disease.

Brauner et al. found that combining the *BRAF* inhibitor and anti-PD-L1 antibody markedly improved tumor immunity (e.g., CD8^+^ cell infiltration and CD8^+^: Treg ratio) and tumor regression in an immunocompetent murine model of ATC. These findings were extended in an immunocompetent model of orthotopic murine ATC by showing that the combination of the *BRAF* inhibitor (i.e., PLX4720) and anti-PD-1/PD-L1 mAbs reduced tumor volume and improved survival [241]. Moreover, the combination of lenvatinib—a multi-tyrosine kinase (TK) inhibitor—with anti-PD-1/PD-L1 mAb reduced tumor volume and improved survival in immunocompetent mice with orthotopic ATC [229]. This combination treatment was associated with an increase in tumor-infiltrating CD8^+^ T cells and granzyme B staining without changes in NK cells. Preliminary studies indicate that the combination of a mAb anti-PD1 (i.e., pembrolizumab) with a TK inhibitor may be an effective salvage therapy for the treatment of ATC [242]. Several ongoing clinical trials are evaluating the strategies that target PD-1 alone or in combination with CTLA-4 inhibitor (ipilimumab), chemotherapy, radiation, or TKI (lenvatinib) in advanced TCs (Table 1). Other clinical trials are evaluating the effects of PD-L1 inhibitors (avelumab, atezolizumab, and durvalumab) in combination with CTLA-4 inhibitor (tremelimumab), chemotherapy, or radiotherapy in advanced TCs (Table 2). Figure 2B schematically illustrates the rationale of combinatorial therapies of advanced TC involving ICIs, BRAFi, multi-targeted TKIs, chemotherapies, or radiotherapies.

## 8. Thyroid Disorders Induced by ICIs

CTLA-4-knockout mice often develop autoimmune diseases, such as pancreatitis and myocarditis [243]. Similar to CTLA-4, mouse models lacking expression of PD-1 have distinct autoimmune phenotypes, such as lupus-like syndromes or dilated cardiomyopathy [244,245]. In humans, inactivating CTLA-4 (i.e., ipilimumab) is linked with a wide array of autoimmune disorders including thyroiditis [246,247,248,249,250,251]. Immune-related adverse events (irAEs) associated with inhibitors of PD-1/PD-L1 axis tend to be more limited in severity and incidence, but have an earlier onset than the adverse effects of CTLA-4 inhibition associated with these agents, which are sometimes severe and can occur during treatment or even long after treatment cessation [230]). Hypothyroidism is more common with anti-PD-1 antibodies than ipilimumab (4–10% versus 2–4% respectively), is rarely severe [252,253,254,255,256,257,258], and occurs commonly after subclinical hyperthyroidism [259,260,261].

## 9. Outstanding Questions and Conclusions

During the last years, the incidence of TC has increased, and it now represents approximately 90% of all endocrine malignancies and 70% of deaths due to endocrine cancers [2]. While the prognosis of DTC is favorable, PDTC and ATC are among the most lethal human malignancies. Nearly all immune cells are present in the TC microenvironment, and in some cases are associated with patient outcome [32,47,121,123,124]. Studies assessing the functional role of TAMs, TAMCs, and TANs have provided evidence for their protumorigenic role [45,47,165]. The majority of these studies were merely quantitative and qualitative analyses of immune cells in the TME of TC. Unfortunately, the presence and functional role(s) of several subsets of immune cells (e.g., NKT and γδ T cells, Tfh, Th9, Th17, and Tc17), which are known to be relevant for tumor initiation and growth, have not yet been investigated in the microenvironment of different types of TC.

Macrophages are key components of the TC microenvironment, and TAM and M2 macrophages are associated with more aggressive cancers (PDTC and ATC) [121], larger tumor size [118], lymph nodes [88], and lung metastases [124]. However, increasing evidence indicates that TAMs comprise more than two (M1 and M2) subsets of cells [116,148]. Mast cell density in human PTC and FTC correlated with tumor extrathyroid extension [47,145]. Mast cells, similar to macrophages, also comprise several subsets of cells [149,150]. Therefore, single-cell RNA-seq will be necessary in order to understand the role of different subsets of macrophages and mast cells in thyroid tumorigenesis and as biomarkers of response to ICIs in patients with advanced disease.

Increasing evidence indicates that neutrophils, which were originally believed to be homogeneous and terminally differentiated cells, are involved in tumor immunity [107,152]. Several studies have simplistically suggested that the NLR could be associated with clinicopathological characteristics of TC patients. The significance of NLR in different types of TC remains uncertain. Galdiero et al. have demonstrated that neutrophils are present in human TC, and TC-CM promoted their activation [165]. Further studies are urgently needed to explore the role of subsets (e.g., low-density, high-density, N1, N2) of neutrophils in different types of TC.

MDSC, similar to macrophages, mast cells, and neutrophils, comprise at least two subsets of cells (i.e., PMN-MDSC and M-MDSC) [170]. PMN-MDSCs in tissues are considered short-living cells, whereas MDSCs in the tumor microenvironment likely represent bona fide M-MDSCs. Unfortunately, the lack of definitive markers for subsets of human MDSCs has so far prevented establishing their exact role in different types of TC. NK cells play a central role in immune surveillance against tumors, and comprise at least two subsets (i.e., CD56^hi^ CD16^lo/hi^ and CD56^lo^ CD16^hi^). In a mouse model of ATC, it was shown that NK cell-based immunotherapy is an effective therapy of pulmonary metastases [192].

TK inhibitors targeting RET or BRAF can induce either stable disease or partial responses in PTC and FTC metastatic patients, but are much less effective in ATC [9,262]. Therefore, alternative therapeutic approaches for these TC histotypes are needed. A possible approach is represented by drugs targeting the interactions between immune cells and cancer cells and/or tumor stromal cells, including immune cells. Various immunologic approaches are under evaluation in preclinical studies or in early phase clinical trials for the treatment of ATC. Preclinical and preliminary clinical studies have reported encouraging results on the efficacy of mAbs targeting the PD-1/PD-L1 network [228]. The rationale of this immunologic approach is based on the expression of the PD-1/PD-L1 pathway in DTC and ATC [237,238]. A promising strategy is the immunotherapy combined or sequenced with targeted therapy in the treatment of tumors [263,264]. Preliminary results suggest that combining BRAF inhibitor and anti-PD-1 mAb can improve immunity in a murine model of ATC [229]. Similarly, the combination of a TK inhibitor with anti-PD-1/PD-L1 mAbs improved survival in a murine model of ATC [229]. Figure 2 schematically illustrates the rationale for the antitumor effect of BRAF or TK inhibitor in combination with ICI.

Several other immune checkpoint receptors such as TIM-3, Lymphocyte-activation gene 3 (LAG-3), T Cell Immunoreceptor With Ig And ITIM Domains (TIGIT), *B- and T-lymphocyte attenuator* (BTLA), V-domain Ig suppressor of T cell activation (VISTA), sialic acid-binding immunoglobulin-type lectins (SIGLEC) 9 and 7, and P-selectin glycoprotein ligand (PSGL)-1 have been identified to be potential therapeutic targets in the immunotherapy of tumors [265]. mAbs targeting the above immune checkpoints are under evaluation in preclinical and/or clinical studies, and should be considered also for the treatment of ATC.

TC cells and several immune cells are a major source of protumorigenic and pro-angiogenic cytokines and chemokines [97,98]. Anti-angiogenic agents, eventually in combination with ICIs, could be exploited to block TC growth, since this strategy has been already developed for other tumors [266]. Moreover, blocking immunosuppressive molecules (TGF-β, IL-10) expressed either by cancer cells or by tumor-infiltrating immune cells [267] could represent another therapeutic strategy for the treatment of TC.

Oncolytic viruses (OVs) are non-pathogenetic viral stains or viral mutants that selectively replicate in and kill tumor cells without causing damage to normal cells [268]. The OV *dl*922-947 reduced CXCL8 and CCL2/MCP-1 expression and inhibited angiogenesis and macrophage infiltration in ATC [269]. The Food and Drug Administration (FDA) has approved the first OV to treat patients with advanced melanoma [270]. Preclinical and clinical studies appear necessary for evaluating ATC virotherapy in the context of TC immunotherapy.

Most of the in vivo experimental studies of TC have been performed with athymic nude mice models. Studies conducted with these models have demonstrated a protumorigenic role of mast cells in human TC [47,48]. The protumorigenic role of macrophages in TC has been established in mice in which macrophage depletion has been obtained by either pharmacological or genetic tools [45,124]. Genetically modified mouse models of TC should be employed to better characterize the role of different immune cell subsets in different stages of tumorigenesis [271].

Extraordinary progress has been made in recent years in the characterization of several, but not all, cells of the immune system in the tumor microenvironment of different TCs. Moreover, it is becoming clear that different subtypes of immune cells play a protumorigenic role, whereas other types play a protective role in TC. Single-cell analysis of peritumoral and intratumoral immune cells could help to elucidate the functions of subsets of cells in different types of TC. The result that will emerge from these studies will contribute to elaborate targeted immunotherapy strategies for the treatment of advanced thyroid cancer.

## Figures and Tables

**Table 1:** Clinical Trials Evaluating the Effects of PD-1 Inhibitors in Thyroid Cancer.

Clinical Trial Registry NCT Number	PD-1 Inhibitor	Combination	Study Phase
NCT03246958	Nivolumab	Nivolumab + Ipilimumab	Phase 2
NCT02834013	Nivolumab	Nivolumab + Ipilimumab	Phase 2
NCT03274258	Nivolumab	Nivolumab + Ipilimumab	Phase 2
NCT03866382	Nivolumab	Nivolumab + Ipilumab	Phase 2
NCT02688608	Pembrolizumab	Pembrolizumab	Phase 2
NCT03072160	Pembrolizumab	Pembrolizumab	Phase 2
NCT03360890	Pembrolizumab	Pembrolizumab + Chemotherapy	Phase 2
NCT03211117	Pembrolizumab	Pembrolizumab + Chemotherapy + Radiation	Phase 2
NCT02973997	Pembrolizumab	Pembrolizumab + Lenvatinib	Phase 2
NCT03012620	Pembrolizumab	Pembrolizumab	Phase 2
NCT03435952	Pembrolizumab	Pembrolizumab + Clostridium Novyi-NT	Phase 1
NCT02628067	Pembrolizumab	Pembrolizumab	Phase 2

**Table 2:** Clinical Trials Evaluating the Effects of PD-L1 Inhibitors in Thyroid Cancer.

NTC Number	PD-L1 Inhibitor	Combination	Study Phase
NCT03181100	Atezolizumab	Atezolizumab + Chemotherapy	Phase 2
NCT03170960	Atezolizumab	Atezolizumab + Cabozantinib	Phase 1 and 2
NCT03217747	Avelumab	Avelumab + Chemotherapy	Phase 1 and 2
NCT03753919	Durvalumab	Durvalumab + Tremelimumab	Phase 2
NCT03215095	Durvalumab	Durvalumab + Radioiodine	Phase 1
NCT03122496	Durvalumab	Durvalumab + Radiotherapy	Phase 1

## Abbreviations

ALK	anaplastic lymphoma kinase
ANGPT	angiopoietin
ATC	anaplastic thyroid carcinoma
CTL	cytotoxic T lymphocyte
CTLA-4	cytotoxic T lymphocyte antigen 4
DCs	dendritic cell
DTC	differentiated thyroid carcinoma
ECM	extracellular matrix
EMT	epithelial-to-mesenchymal transition
FNA	fine-needle aspiration
FTC	follicular thyroid cancer
GM-CSF	granulocyte-macrophage colony-stimulating factor
HGF	hepatocyte growth factor
HTT	hyalinizing trabecular tumor
ICI	immune checkpoint inhibitor
IDO1	indoleamine 2,3-dioxygenase 1
IFN	interferon
ILC	innate lymphoid cell
INF	interferon
mAb	monoclonal antibody
MDSC	myeloid-derived suppressor cell
MMP	metalloproteinase
MNG	multinodular goiter
MTC	medullary thyroid cancer
NGS	next-generation sequencing
NK	natural killer
NKT	natural killer T cell
NLR	neutrophil-to-lymphocyte ratio
NSCLC	non-small-cell lung cancer
OS	overall survival
PD-1	programmed cell death protein-1
PD-L1	programmed cell death ligand-1
PDTC	poorly differentiated thyroid cancer
PMN	polymorphonuclear cell
PTC	papillary thyroid cancer
RET	REarranged during Transfection
RNS	reactive nitrogen species
ROS	reactive oxygen species
SCF	stem cell factor
TAM	tumor-associated macrophage
TAMC	tumor-associated mast cell
TAN	tumor-associated neutrophil
TC	thyroid cancer
TCR	T cell receptor
TDLN	tumor-draining lymph node
Tfh	T follicular helper cell
Th	T helper cell
TK	tyrosine kinase
TKI	tyrosine kinase inhibitor
TME	tumor microenvironment
TNF-α	tumor necrosis factor-α
Treg	regulatory T cell
VEGF	vascular endothelial growth factor
VEGFR	vascular endothelial growth factor receptor
